# Insulin Controls Triacylglycerol Synthesis through Control of Glycerol Metabolism and Despite Increased Lipogenesis

**DOI:** 10.3390/nu11030513

**Published:** 2019-02-28

**Authors:** Ana Cecilia Ho-Palma, Pau Toro, Floriana Rotondo, María del Mar Romero, Marià Alemany, Xavier Remesar, José Antonio Fernández-López

**Affiliations:** 1 Department of Biochemistry and Molecular Biomedicine, Faculty of Biology, University of Barcelona, 08028 Barcelona, Spain; anace.hop@gmail.com (A.C.H.-P.); pautoro_20@hotmail.com (P.T.); floriana.rotondo@gmail.com (F.R.); marromero@ub.edu (M.d.M.R.); malemany@ub.edu (M.A.); xremesar@ub.edu (X.R.); 2 Faculty of Medicine, Universidad Nacional del Centro del Perú, 12006 Huancayo, Perú; 3 Institute of Biomedicine, University of Barcelona, 08028 Barcelona, Spain; 4 Centro de Investigación Biomédica en Red Fisiopatología de la Obesidad y Nutrición (CIBER-OBN), 08028 Barcelona, Spain

**Keywords:** glucose, insulin, lactate, glycerol, adipocytes, lipogenesis

## Abstract

Under normoxic conditions, adipocytes in primary culture convert huge amounts of glucose to lactate and glycerol. This “wasting” of glucose may help to diminish hyperglycemia. Given the importance of insulin in the metabolism, we have studied how it affects adipocyte response to varying glucose levels, and whether the high basal conversion of glucose to 3-carbon fragments is affected by insulin. Rat fat cells were incubated for 24 h in the presence or absence of 175 nM insulin and 3.5, 7, or 14 mM glucose; half of the wells contained ^14^C-glucose. We analyzed glucose label fate, medium metabolites, and the expression of key genes controlling glucose and lipid metabolism. Insulin increased both glucose uptake and the flow of carbon through glycolysis and lipogenesis. Lactate excretion was related to medium glucose levels, which agrees with the purported role of disposing excess (circulating) glucose. When medium glucose was low, most basal glycerol came from lipolysis, but when glucose was high, release of glycerol via breakup of glycerol-3P was predominant. Although insulin promotes lipogenesis, it also limited the synthesis of glycerol-3P from glucose and its incorporation into acyl-glycerols. We assume that this is a mechanism of adipose tissue defense to avoid crippling fat accumulation which has not yet been described.

## 1. Introduction

White adipose tissue (WAT) is a disperse organ [1], distributed in a number of locations in which its basic energy storage activity [2] is complemented by many other physiological functions [3,4,5]. In any case, its main role is to contribute to the defense of energy homoeostasis helping to control glucose [6], lipid [7], and amino acid [8] metabolism. 

Most studies on WAT metabolism have been focused on adipocytes, since they are considered the characteristic and defining cells of WAT. However, adipose tissue structure is far more complex, containing a large number of cell types, not only mature adipocytes, in spite of them occupying most of the space because of their larger size [5]. Between 60 and 80% of the adipose tissue nucleated cells constitute the heterogeneous “stromal vascular fraction”, including stem cells, preadipocytes, endothelial cells, and macrophages [9]. Adipose tissue also contains other cell types such as fibroblasts, histiocytes, mast cells, lymphocytes, granulocytes, blood cells, and nerve terminals. 

An excessive intake of energy may trigger the process that results in chronic insulin resistance and the proinflammatory state that characterizes metabolic syndrome [10]. In all this succession of events, proinflammatory adipokines, released by adipose tissue, play a significant role, as suggested by the fact that the adipose tissue of obese individuals expresses proinflammatory adipokines in a higher proportion than in those with normal weight [11]. Proinflammatory effects are not limited to increased circulating adipokines released by adipose tissue; excess nutrients such as circulating lipids or glucose may become akin to “toxic”, enhancing oxidative stress and promoting inflammatory responses, whereas food restriction to normal levels reduces both [12].

We recently found that, under normoxic conditions, adipocytes in primary cultures were able to convert huge amounts of the medium glucose to lactate and glycerol, fulfilling most of their energy needs through anaerobic glycolysis [13,14]. We postulated that this “wasting” of glucose might help to diminish hyperglycemia because of the large combined mass of WAT. In *ex vivo* studies, we also observed the accumulation of lactate in WAT masses [15], in agreement with the results observed in cultured cells and the low *in vivo* WAT oxygen consumption [16,17,18] and high WAT lactate production [18,19] observed in humans.

The “unnecessary” anaerobic use of glucose by adipocytes (and WAT as a whole) producing large amounts of 3-carbon fragments seems to be intrinsic to the tissue, because the high production of lactate occurred in the absence of external stimuli, and independent of the availability of oxygen. Given the assumed importance of insulin in the metabolism of adipose tissue, and because of its anabolic role, we wanted to discern whether insulin could indeed favor the accumulation of triacylglycerol (TAG) reserves in the adipocyte, since this way it would hinder the breakup of excess glucose to 3C fragments. The consequence could be the loss of the potential protective action of the tissue modulating hyperglycemia. Consequently, we investigated how insulin intervened on glucose disposal via glycolysis to 3C fragments, in contrast to its effect on fatty acid synthesis and increased TAG storage. We quantitatively analyzed the fate of labelled glucose and the metabolic changes induced by insulin on primary cultures of adipocytes. Quantitative analyses of metabolites and key gene expressions allowed us to obtain a wider picture of what is in fact the actual role of insulin in the handling of glucose loads by mature adipocytes.

## 2. Materials and Methods 

### 2.1. Rats and Sampling

All animal handling procedures and the experimental setup were in accordance with the animal handling guidelines of the corresponding European and Catalan Authorities. The Committee on Animal Experimentation of the University of Barcelona specifically authorized the specific procedures used (# DAAM 6911).

Healthy adult male Wistar rats (Janvier, Le Genest-Saint Isle, France), weighing 399 ± 64 g were used. The animals were kept in two-rat cages with wood shards as bedding material, at 21–22 °C, and 50–60% relative humidity; lights were on from 08:00 to 20:00. The rats had unrestricted access to water and standard rat chow (#2014, Teklad Diets, Madison, WI, USA).

The rats were sacrificed, under isoflurane anesthesia, by exsanguination from the exposed aorta. They were dissected and samples of epididymal WAT were extracted and minced with scissors before further processing.

### 2.2. WAT Adipocyte Isolation and Incubation Procedures

Cells were isolated [20] at 37 °C for 1 h in a shaking bath using collagenase (LS004196, type I, from Worthington Biomedical, Lakewood, NJ, USA) in 2.5 volumes of modified Krebs–Henseleit buffer [21]. The cell suspension was filtered through a double layer of nylon hose, transferred to vertical syringes, and left standing for 5–6 min at room temperature. Adipocytes formed an upper loose cake, floating over a liquid phase that was slowly drained from the syringe; the adipocyte layer was gently re-suspended in fresh buffer (free of collagenase) and the process of mixing and draining was repeated twice, discarding the washing fluids. Aliquots of the adipocyte layer were used for incubation immediately after the final washing. All cell preparations were maintained at room temperature (approximately 22 °C), and manipulated within a time as short as possible. Cells were counted and their spherical (when free) diameters measured using ImageJ software (http://imagej.nih.gov/ij/).

The complete cell incubation procedure was previously described by us [14,22]. Briefly, cell incubations were carried out using 12-well plates (#734-2324VWR International BVBA/Sprl., Leuven, Belgium) filled with 1.7 mL of DMEM (#11966-DMEM-no glucose; Gibco, Thermo-Fisher Scientific, Waltham, MA, USA), supplemented with 30 mL/L fetal bovine serum (FBS, Gibco). The medium also contained 25 mM HEPES (Sigma-Aldrich, St Louis, MO, USA), 2mM glutamine (Lonza Biowhittaker, Radnor, PA, USA), 30 mg/mL delipidated bovine serum albumin (Millipore Calbiochem, Bedford, MA, USA), and 100 nM adenosine, 100 U/mL penicillin plus 100 mg/L streptomycin (Sigma-Aldrich). Half of the wells were supplemented with bovine insulin (Sigma-Aldrich): final concentration 175 nM.

The incubation medium was also supplemented with ^14^C-(U)-D glucose, (#ARC0122B, American Radiolabeled Chemicals, St. Louis, MO, USA; specific radioactivity 11 GBq/mmol). Final glucose concentrations in the wells were, nominally, 3.5, 7, or 14 mM. In the labelled samples, the amount of label added per well was approximately 394 Bq/mmol of glucose.

A “parallel” series of wells was developed, containing the same adipocytes’ suspension and identical medium composition and other conditions than those described above, but in which no label was added. These wells were used for cell gene transcription and medium metabolite analyses.

Each well received 400 μL of the corresponding cell suspension; after initial sampling (100 µL), the final incubation volume was 2.0 mL. The cells were incubated at 37 °C in a chamber ventilated with air supplemented with 5% CO_2_, which gave a theoretical pO_2_ of 20 kPa [23]. The cells were incubated for 24 h without any further intervention.

### 2.3. Processing of the Incubation Media: Label Distribution

The label-containing samples were used to fraction the label distribution applying a protocol previously described by us [13]. Lactate (including pyruvate) label was determined using centrifuge microcolumns made up with sieve-filter type centrifugation inserts (Ultrafree-MC, Millipore, Bedford, MA, USA) containing 250 mg of hydrated, spin-dried cationic-form Dowex 1 × 2 ion exchange resin (Sigma-Aldrich) [13]. The retained lactate was eluted with acid and counted.

The medium free of lactate was used in part to convert all glucose to gluconate by incubation with glucose oxidase (type VII from *Aspergillus niger*, Sigma-Aldrich); as well as catalase (from bovine liver, Sigma-Aldrich). Catalase was added to destroy H_2_O_2_ and to help maintain O_2_ availability. The change of nonionic glucose to gluconate allowed its retention (and acidic elution) using microcolumns as described for lactate. The label retained was that of the unaltered glucose remaining in the medium after incubation [13,24].

A second aliquot of the label-containing medium free of lactate was treated with glycerol kinase (from *Escherichia coli*, #G6278, Sigma-Aldrich) and ATP for the conversion of glycerol to glycerol-3P. The change in ionization was used to remove the glycerol (as glycerol-3P) from the medium, eluting it with acid and thus counting the label retained in the glycerol moiety [13,25].

Combination of “cold” metabolite measurements and their radioactivity allowed us to calculate the fate of the initial glucose label under all conditions tested and to estimate the specific-C radioactivity for all of them.

Carbon dioxide production along the lipogenic process was estimated from the label present in the TAG fatty acids. We considered that for every 3C from glucose, one is lost in the conversion to 2C fragments (from pyruvate to acetyl), and assumed that 1 mole of CO_2_ was produced in the pentose-P pathway for each 2 moles of NADPH generated (explained in more detail in Ho-Palma et al. [13]). This amount of CO_2_ represents the minimum possible values, since the CO_2_ that could have been produced in oxidative processes is not included.

### 2.4. Processing of Labelled Cell Components

The procedure for measuring label distribution in the different cell fractions and media has been previously developed, tested, and quantified [13]. Briefly, the cells incubated with labelled glucose were weighed, frozen with liquid nitrogen, transferred to glass tubes, and immediately extracted with chilled peroxide-free diethyl ether [26]. The aqueous fraction (and interface) was wholly used to estimate radioactivity. It contained mainly glycogen, but also cell proteins, metabolites, membrane microsomes, and non-lipophilic debris. The organic phase, containing essentially TAG, was dried, weighed, re-dissolved in ethyl ether, and saponified with KOH in ethanol in the cold. The ether-insoluble potassium soaps were extracted and counted. The aqueous phase, containing all glycerides-glycerol, was also removed and counted [13]. Soap label was that of TAG fatty acids. 

### 2.5. Analysis of Metabolites in the Medium

Medium glucose was measured using a glucose oxidase-peroxidase kit (#11504, Biosystems, Barcelona, Spain) to which we added 740 nkat/mL mutarotase (porcine kidney, 136A5000, Calzyme, St. Louis, MO, USA) [27]. Lactate was measured with kit 1001330 (Spinreact, Sant Esteve d’en Bas, Spain); glycerol was estimated with kit #F6428 (Sigma-Aldrich). Non-esterified fatty acids (NEFA) were measured using kit NEFA-HR (Wako Life Sciences, Mountain View, CA, USA). 

### 2.6. Gene Expression Analysis

Total cell RNA was extracted from all the harvested cells (parallel, non-labelled plates) using the Tripure reagent (Roche Applied Science, Indianapolis, IN, USA). RNA content was quantified in an ND-1000 spectrophotometer (NanoDrop Technologies, Wilmington, DE, USA). RNA samples were reverse transcribed using oligo-dT primers (Gene Link, Westchester, NY, USA) and the MMLV reverse transcriptase (Promega, Madison, WI, USA) system.

Real-time PCR amplification was carried out using 10 μL amplification mixtures containing Power SYBR Green PCR Master Mix (Applied Biosystems, Foster City, CA, USA), 4 ng of reverse-transcribed RNA, and 150 nmol of primers. Reactions were run on an ABI PRISM 7900 HT detection system (Applied Biosystems) using a fluorescent threshold manually set to 0.5 for all runs.

A semi-quantitative approach for the estimation of the concentration of specific gene mRNAs per unit of tissue weight was used [28]. *Arbp* was the charge control gene [29]. We expressed the data as the number of transcript copies per adipocyte. The genes analyzed and a list of primers used are presented in Table 1.

### 2.7. Statistical Procedures

Statistical analyses and the establishment of significant differences between groups (two-way ANOVAs and post-hoc Sidak test) were done with the GraphPad Prism 6 program (GraphPad Software, La Jolla, CA, USA). 

## 3. Results

**Figure 1:**
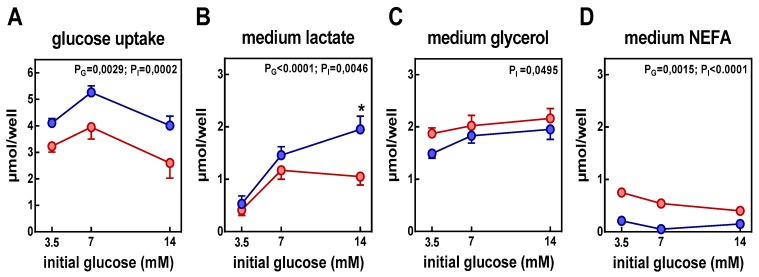
Effect of insulin on the rates of glucose uptake and other metabolite efflux after the incubation of adipocytes for 24 h with 3.5, 7, or 14 mM glucose. The data are the mean ± SEM from 12 different animals. Values are expressed as µmol per well of (**A**) glucose taken, or (**B**) lactate, (**C**) glycerol, and (**D**) NEFA (non-esterified fatty acids) released to the medium after 24 h of incubation. Red circles: adipocytes incubated in absence of insulin; blue circles: adipocytes incubated in presence of insulin. Statistical significance of the differences between groups (2-way ANOVA): P_G_ corresponds to the differences with respect to initial glucose, and P_I_ corresponds to the differences with respect to the presence or not of insulin. Not significant values (*p* > 0.05) were not represented. *post-hoc significant differences between groups with and without insulin of identical glucose concentration.

Figure 1 shows the rates of glucose uptake and other metabolite efflux after the incubation of adipocytes for 24 h with 3.5, 7, or 14 mM glucose, in the presence or absence of insulin. Glucose uptake by adipocytes was slightly affected by initial glucose concentration showing a peak with 7 mM initial glucose. Lactate efflux was also affected by initial glucose concentration, being its efflux greater at 7 and 14 mM glucose; conversely, NEFA efflux decreased with glucose concentration. Insulin, increased lactate efflux, slightly decreased that of glycerol, and almost abolished NEFA output in adipocytes.

**Figure 2:**
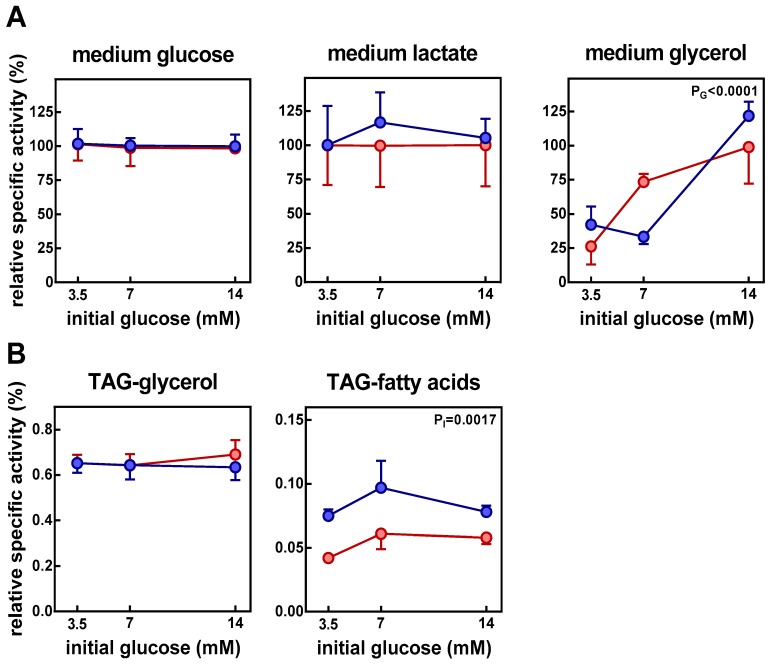
Insulin effect in the carbon specific radioactivity of the main label fractions obtained from ^14^C-glucose after incubation of epididymal adipocytes in a primary culture. The data are presented as mean ± SEM from six different animals. Specific radioactivities (**A**) from label fractions (glucose, lactate, and glycerol) in the medium and (**B**) from label fractions (glycerol and fatty acids) in cell triacylglycerol (TAG). C-specific radioactivity corresponds to the quotient of label found in the fraction divided by the molar concentration and the number of carbons the compound contains. In this case, all data have been referred to initial glucose C-specific radioactivity, to which a value of 100 was given. Red circles: adipocytes incubated in absence of insulin; blue circles: adipocytes incubated in presence of insulin. The statistical significance data and conventions are the same as in Figure 1.

Figure 2 shows the specific carbon radioactivities of the fractions in which direct measurement of cold and labelled compounds were done. The data are shown as percentages of the initial glucose carbon specific activity. As expected, glucose specific activity was maintained independently of insulin or initial glucose concentration; that of lactate was also maintained on the same range than glucose. In adipocytes, specific radioactivities of free glycerol increased with the amount of glucose in the medium: with elevated concentrations of glucose (14 mM initial glucose), most of the glycerol was obtained from glucose; with lower glucose concentrations, a progressively smaller proportion of glycerol had its origin from glucose.

In adipocytes, the specific activity of glycerides-glycerol was low, and was unaffected by the initial glucose concentration or the presence of insulin. The specific radioactivity of the glycerides-fatty acids was very low, close to one order of magnitude lower than that of glycerol in glycerides, although in this case the presence of insulin increased specific radioactivity.

**Figure 3:**
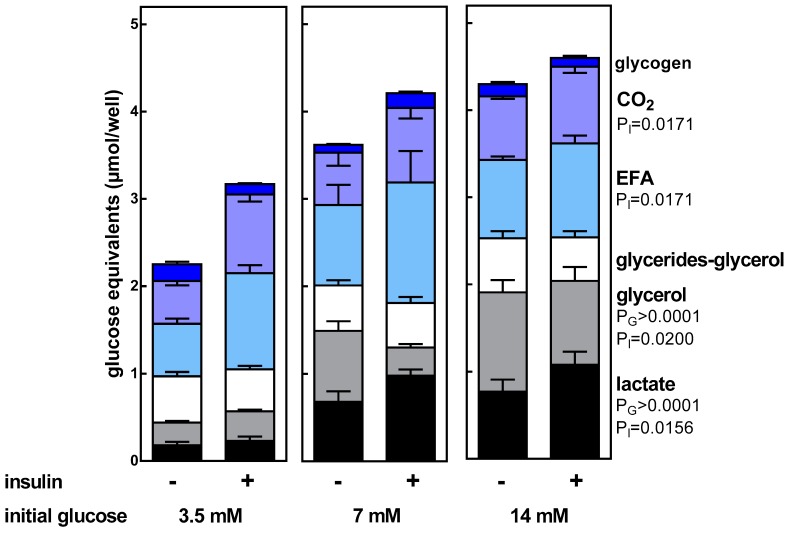
Final distribution of the medium glucose label calculated from the radioactivity incorporated into the different label-containing fractions studied. Lactate and glycerol data correspond to the amount of lactate and glycerol found in the medium; glycerides-glycerol, EFA, and glycogen data were obtained from the cell intracellular content; CO_2_ was calculated from EFA values as indicated in Materials and Methods. The data (calculated as glucose equivalents) are presented as mean ± SEM from six different animals. EFA = esterified fatty acids (in the cell lipid droplet). The statistical significance data are the same as in Figure 1. Since CO_2_ was directly calculated from the radioactivity found in the esterified fatty acids, the statistical data were similar to that of EFA.

Figure 3 shows the main metabolites obtained from glucose metabolism. The major products, at least with high glucose concentrations, were lactate and glycerol. In the case of glycerol, the results were different from those obtained in Figure 1, since glycerol production from glucose and its release to the medium increased with the concentration of glucose; however, glycerol (from glucose) incorporated into TAG was constant and independent of both glucose concentration and insulin. On the contrary, insulin stimulated the synthesis of lactate and fatty acids (and consequently CO_2_) from glucose. Glucose incorporation into glycogen was small and unaffected by insulin.

**Figure 4:**
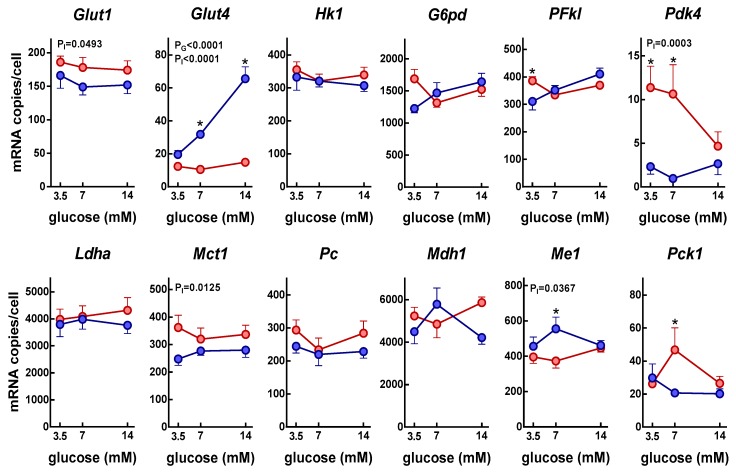
Insulin effect on the gene expressions of proteins related to glucose metabolism in adipocytes incubated under varying glucose concentrations for 24 h. The data are presented as number of the corresponding mRNA copies per cell, and are mean ± SEM of data from six rats. The data were obtained from the “parallel” incubations (i.e., no label). Red circles: adipocytes incubated in absence of insulin; blue circles: adipocytes incubated in presence of insulin. The statistical significance data and conventions are the same as in Figure 1. *post-hoc significant differences between groups with and without insulin of identical glucose concentration. The correspondence between gene names and those of the proteins they code can be seen in Table 1.

Figure 4 presents the changes in gene expression of key enzymes and transporters implicated in the glycolytic utilization of glucose by adipocytes. Data were expressed as the number of copies of the corresponding mRNA per cell. Each well contained 6.32 ± 0.14 × 10^5^ adipocytes (i.e., 0.34 g of adipose tissue). The glucose transporter gene *Glut1*, showed a similar number of copies of its mRNA per cell for all glucose concentration groups, but insulin decreased its expression. The pattern for glucose transporter gene *Glut4* was different, since both insulin and glucose increased its expression; in the case of glucose, the increase only occurred in the presence of insulin. Expression levels of *Glut4* were lower than those of *Glut1*.

Expressions of hexokinase (*Hk1*), 6-phosphofructokinase liver type (*Pfkl*), lactate dehydrogenase (*Ldha*), pyruvate carboxylase (*Pc*), and malate dehydrogenase (*Mdh1*) were unaffected by glucose concentration and insulin. The monocarboxylate transporter gene (*Mct1*), responsible for lactate (and pyruvate) efflux, slightly decreased its expression in the presence of insulin.

The expression of phospho-enolpyruvate carboxykinase (*Pck1*), a main control point for the regulation of gluconeogenesis in liver, was lower in the presence of insulin, but only at 7 mM glucose.

The data suggest that insulin increased the activity of pyruvate dehydrogenase, the enzyme responsible for the conversion of pyruvate to acetyl-CoA, by decreasing the expression of its main inhibitor, pyruvate dehydrogenase kinase 4 (*Pdk4*).

The main providers of NADPH in the cytoplasm to sustain lipogenesis are malic enzyme and the reductive part of the pentose-phosphate cycle. There was a tendency to increase the expression of *Me1* but not *G6pd* by insulin.

**Figure 5:**
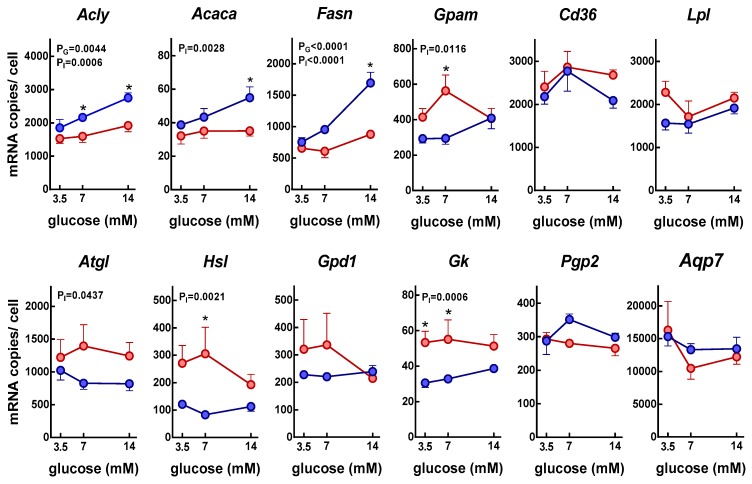
Insulin effect in gene expression of proteins related to lipids and glycerol metabolism in adipocytes incubated under varying glucose concentrations for 24 h. The data are presented as number of the corresponding mRNA copies per cell, and are mean ± SEM of data from six rats. The data were obtained from the “parallel” incubations (i.e., no label). Red circles: adipocytes incubated in absence of insulin; blue circles: adipocytes incubated in presence of insulin. The statistical significance data and conventions are the same as in Figure 1 and Figure 4. The correspondence between gene names and those of the proteins they code can be seen in Table 1. *post-hoc significant differences between groups with and without insulin of identical glucose concentration.

Figure 5 shows the changes in gene expression of key enzymes and transporters implicated in the glycerol and lipid metabolism of adipocytes. When we analyzed the expression of three key points of control of lipogenesis, ATP: citrate lyase (*Acly*), acetyl-CoA carboxylase (*Acaca*), and fatty acid synthase (*Fas*) genes, a similar profile was observed, with higher expressions in the presence of insulin and higher glucose concentrations. This latter effect was only apparent in the presence of both glucose and insulin. Nevertheless, the expression of glycerol-3P acyl-transferase (*Gpam*), a critical enzyme for TAG synthesis, was inhibited by insulin.

The expression of the main enzymes responsible for endogenous lipolysis (triacylglycerol lipase, *Atgl*, and hormone-sensitive lipase, *Hsl*) was inhibited by insulin. In the case of lipoprotein lipase (*Lpl*) responsible for the degradation of triacylglycerols of exogenous origin, it was not affected by glucose or insulin.

The expression of the enzymes responsible for the synthesis of glycerol 3-phosphate in the adipocyte tended to be inhibited by the presence of insulin. This was the case of the glyceraldehyde-3-phosphate dehydrogenase (*Gdp1*; although the inhibition was not statistically significant, *p* = 0.0628), responsible for the synthesis of glycerol 3-phosphate from dihydroxyacetone-phosphate, and that of glycerokinase (*Gk*) responsible for the phosphorylation of glycerol. The expression of this last enzyme was very low. In the case of the enzymes that use glycerol-3-phosphate as substrate, the expression of phosphoglycolate phosphatase (*Pgp2*), which catalyzes the hydrolysis of glycerol 3-phosphate to glycerol, was not affected.

Finally, the expression of aquaporin 7, a transporter playing an important role in glycerol transport, and *Cd36*, that imports fatty acids inside cells, was unaffected by the conditions tested in our study.

## 4. Discussion

Adipose tissue is a complex organ, made up of different tissue masses. One of the limitations of the study was that only adipocytes of epididymal origin were used. Previous studies indicate the existence of differences in the amount of lactate and glycerol produced by adipocytes from different locations, as well as in the expression of genes related to both carbohydrate and lipid metabolism, an aspect mainly affected by sex [30,31]. In any case, adipose tissues from all sites seem to share a similar metabolic profile, only showing differences that are mainly quantitative.

Under basal conditions, and in the absence of insulin, isolated adipocytes and intact WAT use significant amounts of glucose and secrete high amounts of 3C metabolites as lactate and glycerol. These 3C compounds may be used for hepatic gluconeogenesis [32] or lipogenesis [33], or for energy purposes in other tissues. 

WAT limits excessive TAG accumulation (excess energy) in the tissue, reducing substrate availability by decreasing blood flow [34]. Additionally, with this high production of lactate and glycerol release (i.e., glucose breakup), WAT defends itself from excess glucose, preventing an inordinate enlargement of its TAG stores [23]. The utilization of 3C metabolites by tissues is unaffected by insulin resistance and circumvents the strict regulation of glycolysis, providing partially metabolized and directly usable energy substrates. The results presented here agree with this interpretation, confirming that most of the glucose taken up by adipocytes is just returned to the medium as 3C metabolites, essentially lactate and glycerol [35], thus helping to lower circulating glucose availability. 

**Figure 6:**
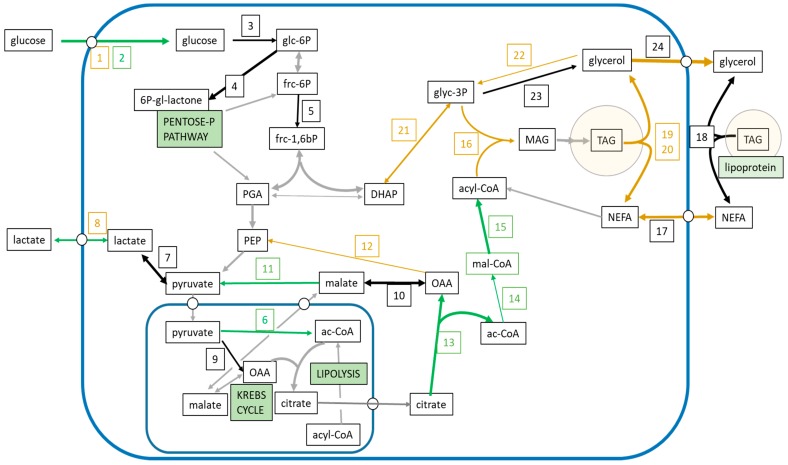
Insulin regulation of the main metabolic pathways in the adipocyte during incubation with glucose. The graph presents the main intermediate metabolites and substrates. Green lines correspond to pathways activated by incubation with insulin, whereas brown lines represent inhibited pathways. The figure incorporates data from label fate, metabolite concentrations, specific radioactivity, and gene expression. The squares with numbers represent the genes whose expression has been measured. 1—*Glut1* (glucose transporter type 1). 2—*Glut4* (glucose transporter type 4). *3*—*Hk1* (hexokinase 1). 4—*G6pd* (glucose-6-phosphate dehydrogenase). 5—*Pfkl* (phospho-fructokinase, liver, b-type). 6—*Pdh* (pyruvate dehydrogenase) (activation resulting from marked inhibition of the expression of *Pdk4*, pyruvate dehydrogenase kinase 4). 7—*Ldha* (L-lactate dehydrogenase a). 8—*Mct1* (monocarboxylate transporter 1). 9—*Pc* (pyruvate carboxylase). 10—*Mdh1* (malate dehydrogenase). 11—*Me1* (malic enzyme). 12—*Pck1* (phospho-enolpyruvate carboxykinase). 13—*Acly* (ATP: citrate lyase). 14—*Acaca* (acetyl-CoA carboxylase alpha). 15—*Fas* (fatty acid synthase). 16—*Gpam* (glycerol-3P acyl-transferase). 17—*Cd36* (platelet glycoprotein 4) (fatty acid transporter). 18—*Lpl* (lipoprotein lipase). 19—*Atgl* (triacylglycerol lipase, adipose tissue). 20—*Hsl* (hormone-sensitive lipase). 21—*Gdp1* (glycerol-3P dehydrogenase). 22—*Gk* (glycerol kinase). 23—*Pgp2* (phospho-glycolate phosphatase). 24—*Aqp7* (aquoporin 7). 6P-gl-lactone = 6-phosphogluconolactone; ac-CoA = acetyl coenzyme A; DHAP = dihydroxyacetone phosphate; frc-1,6bP = fructose 1,6-bisphosphate; frc-6P = fructose 6-phosphate; glc-6P =glucose 6-phosphate; glyc-3P = glycerol 3-phosphate; MAG = monoacylglycerol; mal-CoA = malonyl coenzyme A; NEFA = non-esterified fatty acids; PEP = phosphoenolpyruvate; PGA = 3-phosphoglycerate; OAA = oxaloacetate; TAG = triacylglycerol.

As expected, insulin elicited an anabolic response in WAT, increasing glucose uptake [36], as well as the flow of carbon through glycolysis and lipogenesis [37]. Figure 6 shows the actions of insulin on these main metabolic pathways in the context of the glucose-fatty acid metabolism in the adipocyte.

Basal glucose uptake was high, even in the absence of insulin, mainly due to the high expression of *Glut1*, a widely distributed insulin-independent glucose transporter. Insulin increased glucose uptake, despite a lower expression of *Glut1*, compensated by a higher expression of *Glut4*, which facilitates glucose transport into insulin-sensitive cells. *Glut4* is responsible for the insulin-regulated glucose transport into muscle and adipose cells [38], but a large proportion of glucose enters most body cells by means of *Glut1*. This is the most widely expressed hexose transporter, whose main role is assumed to maintain basal glucose transport in most types of cells [39,40]. *Glut1* is regulated through the control of gene expression [41,42,43], and only in part via regulative control, which is the main control system for *Glut4* [44].

It has been described that hypoxia increases monosaccharide uptake capacity in adipocytes, contributing to adipose tissue dysregulation in obesity [45]. However, the effects on glucose transporters are the opposite of those induced by insulin: exposure of adipocytes to hypoxia and increased *Glut1* expression, in contrast with no changes in *Glut4* [45].

Although there were no changes in the expression of genes related to the mainstream glycolysis (such as hexokinase and phosphofructokinases), there was a clear increase in the flow of carbon along the glycolytic pathway, as shown by the increased glucose uptake of cultured adipocytes and higher lactate efflux to the medium. The absence of changes in the expression of phosphofructokinases, one of the key regulatory and rate limiting steps of glycolysis, may be explained in part because phosphofructokinase regulation of glycolysis is essentially done through allosteric mechanisms [46].

Lactate and glycerol production (from glucose) helps sustain the basic energy needs of the cell through a fully anaerobic (albeit facultative, since it occurs under normoxic conditions) process [23]. The sheer size of adipocytes, and thin cytoplasm layer around its huge lipid vacuole, hamper the intracellular circulation of substrates, thus limiting and compartmentalizing most metabolic activities. In adipose tissue, glucose can be taken up easily from the circulation by adipocytes and converted anaerobically to lactate, pyruvate, or glycerol, covering the minimal needs of ATP. However, the production of acetyl-CoA requires access to mitochondria, few and sparsely distributed in large adipocytes [47]. Additionally, glucose utilization helps lower glycaemia, breaking up 6C to 3C molecules. This way, a portion of circulating glucose was substituted in large proportions by less-regulated 3C substrates used elsewhere for energy but also for splanchnic lipogenesis or gluconeogenesis. Therefore, WAT lactate efflux may be considered primarily a normal consequence of the need to use glycolytic (anaerobic) ATP and to eliminate excess circulating glucose. Consequently, it should not be considered a specific indicator of hypoxia, despite its generalized association with lactate [48]. This is reinforced by the fact that the high production of lactate (and that of glycerol) from glucose only occurs in the presence of high concentrations of glucose (in our case, 7 and 14 mM), although glucose uptake by the adipocyte is similar in all cases. Glucose is used almost entirely anaerobically at 14 mM glucose concentrations. Accordingly, higher circulating levels of lactate and glycerol are observed in type 2 diabetes [49]; likewise, obese women with higher glucose levels after an oral glucose tolerance test, release more glycerol and lactate from their subcutaneous adipose tissue, both in the postabsorptive state and after the oral glucose tolerance test [50].

On the other hand, the global production of glycerol was preserved and sustained, remaining practically constant regardless of the medium initial glucose concentration. This maintained production of glycerol by adipocytes in amounts similar or even greater than those of lactate, together with the different origin of glycerol according to the availability of glucose in the medium, suggest that glycerol release represents a constitutive process rather independent of external factors. Insulin only slightly decreased glycerol release to the medium, probably a consequence of the inhibition of lipolysis by insulin. In any case, the limitation of lipolysis by insulin was minimal (at the limit of significance in Figure 1C, and absent in Figure 2B) under the *in vitro* conditions tested. This may be due to the absence of any previous stimulation of lipolysis and/or to the presence of adenosine (a known inhibitor of lipolysis [51]) in the medium. Glycerol-3P, derived from glucose via trioses-P, is hydrolyzed to glycerol by a glycerophosphatase, as previously postulated by us [52]. This phosphatase, recently described in liver, is also present in WAT [53]. The expression of this enzyme (*Pgp2*) was unaffected by both glucose and insulin and was also not correlated with the lower efflux of glycerol observed in insulin-treated cells. The removal of excess glycerol-3-P under heavy glycolytic pressure (high glucose levels, insulin activation of *Glut4*) may help lower its cell pool, thus helping to maintain a lower glycerol esterification with acyl-CoA and thus downregulate TAG synthesis. 

The equilibrium between these pathways—(a) incorporation into the acyl-glycerol pool or (b) direct generation of glycerol from glycerol-3P—seems to be a critical point in the regulation of TAG synthesis, regulated by insulin, which lowers the expression of glycerol-P acyl-transferase despite a marked increase in gene expressions along the lipogenic pathway. Glycerol-3-phosphate acyltransferase is the rate-limiting enzyme in the glycerolipid synthesis pathway [54]. Thus, this critically different regulation of glycerol-P acyl-transferase expression, with respect to the other lipogenic enzymes, can be tentatively explained because the induction of glycerol-P acyl-transferase mRNA by insulin is mediated through the expression of SREBP-1, independently of ChREBP [55,56]. Insulin increases SREBP-1 expression in liver and adipose tissue, but only increases the post-translational activation of SREBP-1c in liver, decreasing it in adipose tissue [57]; consequently, it inhibits the expression of adipose glycerol-P acyl-transferase.

The production of glycerol is more complex than that of lactate. Although its contribution to 3C fragment production from glucose and lowering glycaemia may also be accomplished by lactate, glycerol synthesis is nevertheless preferred. These data agree with our previous findings indicating that adipocytes’ glycerol production is a definite objective in addition to being a 3C substrate [14].

The lower production of glycerol and, therefore, the attempt to limit the accumulation of lipid in the adipocyte, has an obvious protective effect for the adipocyte itself. It can be speculated that this action, in contrast, could be counterproductive for the organism, since it may favor the ectopic deposition of lipid in other tissues. However, fat content in all WAT depots change in a proportion similar to that of the lipids in the rest of organs and tissues [2], which makes this possibility highly improbable. Similarly, although adipocytes may potentially decrease blood glucose levels by releasing lactate, it has been described that high hepatic lactate levels favor the appearance of NASH [58]. Again, a protective effect in the tissue could have unwanted consequences favoring the development of metabolic syndrome-related pathologies.

The higher incorporation of the glucose carbons into fatty acids of the TAG in the presence of insulin, but without changes in the incorporation of glycerol into the TAG, suggests that insulin induces a higher turnover of TAG. However, we cannot distinguish whether the origin of these TAG is endogenous or exogenous, from the action of the LPL on the adipocyte debris present in the medium. The activation of TAG turnover [14], when glycerogenic flow decreases, is contrary to the assumed focus of insulin-stimulated adipocytes, where lipogenesis and inhibition of lipolysis are trends clearly established through gene expression patterns.

## 5. Conclusions

In conclusion, under conditions of high availability of glucose and energy, WAT (at least the adipocytes) defend themselves to prevent the pathologic accumulation of excess energy. Part of the panoply of adipose tissue defense systems to prevent this damage include the following: (a) lower blood flow through the tissue, to decrease the arrival of nutrients [34]; (b) the adipocyte massively metabolizes excess glucose to fragments of three carbons that can be used for energy elsewhere, but also helping to decrease hyperglycemia; (c) WAT, especially under conditions of obesity, is able to inactivate large amounts of insulin [59], thus limiting its lipogenic effect; and (d) last, but not least, as we have shown here, insulin priority rests on the derivation of excess C to the secretion of glycerol rather than on a higher incorporation of glycerol-3P into the synthesis of TAG. In all cases, the measures are short term, and may have only a limited success since, in any case, the presence of insulin induces a higher incorporation of glucose carbon into the adipocyte TAG pool.

## Figures and Tables

**Table 1:** **Table 1**. Primers used for the analysis of gene expression.

	Protein	5′ primer	3′ primer	bp
*Glut1*	glucose transporter type 1	GCTCGGGTATCGTCAACACG	ATGCCAGCCAGACCAATGAG	97
*Glut4*	glucose transporter type 4	CTTGATGACGGTGGCTCTGC	CACAATGAACCAGGGGATGG	127
*Hk1*	hexokinase 1	TGGATGGGACGCTCTACAAA	GACAGGAGGAAGGACACGGTA	100
*G6pd*	glucose-6-phosphate dehydrogenase	GACTGTGGGCAAGCTCCTCAA	GCTAGTGTGGCTATGGGCAGGT	77
*PFkL*	phospho-fructokinase, liver, b-type	CAGCCACCATCAGCAACAAT	TGCGGTCACAACTCTCCATT	90
*Pdk4*	pyruvate dehydrogenase kinase 4	CTGCTCCAACGCCTGTGAT	GCATCTGTCCCATAGCCTGA	142
*Ldha*	L-lactate dehydrogenase a	AAAGGCTGGGAGTTCATCCA	CGGCGACATTCACACCACT	96
*Mct1*	monocarboxylate transporter 1	CCCAGAGGTTCTCCAGTGCT	ACGCCACAAGCCCAGTATGT	133
*Pc*	pyruvate carboxylase	GCCAGAGGCAGGTGTTCTTTG	TTTGGCCCTTCACATCCTTCA	120
*Mdh1*	malate dehydrogenase 1	GCTGGCTCAAGGGAGAGTTC	TCTCATGTGGTCCGAGATGG	116
*Me1*	NADP^+^-dependent malic enzyme	GGAGTTGCTCTTGGGGTAGTGG	CGGATGGTGTTCAAAGGAGGA	143
*Pck1*	phosphoenolpyruvate carboxykinase 1	CGGGTGGAAAGTTGAATGTG	AATGGCGTTCGGATTTGTCT	142
*Acly*	ATP citrate lyase	TGTGCTGGGAAGGAGTATGG	GCTGCTGGCTCGGTTACAT	137
*Acaca*	acetyl-coA carboxylase alpha	AGGAAGATGGTGTCCGCTCTG	GGGGAGATGTGCTGGGTCAT	145
*Fasn*	fatty acid synthase	CCCGTTGGAGGTGTCTTCA	AAGGTTCAGGGTGCCATTGT	117
*Gpam*	glycerol-3P acyl-transferase	GGTGAGGAGCAGCGTGATT	GTGGACAAAGATGGCAGCAG	129
*Cd36*	platelet glycoprotein 4 (fatty acid transporter)	TGGTCCCAGTCTCATTTAGCC	TTGGATGTGGAACCCATAACTG	154
*Lpl*	lipoprotein lipase	TGGCGTGGCAGGAAGTCT	CCGCATCATCAGGAGAAAGG	116
*Atgl*	triacylglycerol lipase (adipose tissue)	CACCAACACCAGCATCCAAT	CGAAGTCCATCTCGGTAGCC	120
*Hsl*	hormone-sensitive lipase	TCCTCTGCTTCTCCCTCTCG	ATGGTCCTCCGTCTCTGTCC	108
*Gpd1*	glycerol-3P dehydrogenase (NAD^+^)	CTGGAGAAAGAGATGCTGAACG	GCGGTGAACAAGGGAAACTT	113
*Gk*	glycerol kinase	ACTTGGCAGAGACAAACCTGTG	ACCAGCGGATTACAGCACCA	74
*Pgp2*	phosphoglycolate phosphatase (glycerophosphatase)	CCTGGACACAGACATCCTCCT	TTCCTGATTGCTCTTCACATCC	100
*Aqp7*	aquaporin 7	ACAGGTCCCAAATCCACTGC	CCGTGATGGCGAAGATACAC	127
*Arbp*	0 S acidic ribosomal phosphoprotein PO (housekeeping gene)	CCTTCTCCTTCGGGCTGAT	CACATTGCGGACACCCTCTA	122
